# Evaluating Gait Abnormalities in Asian Elephants Using Inertial Measurement Unit-Based Vertical Movement Symmetry Analysis: A Pilot Study

**DOI:** 10.3390/vetsci12020154

**Published:** 2025-02-11

**Authors:** Siripat Khammesri, Kittichai Wantanajittikul, Kittikul Namwongprom, Narueporn Kittisirikul, Pichamon Ueangpaibool, Chatchote Thitaram, Janine L. Brown, Siriphan Kongsawasdi

**Affiliations:** 1 Department of Physical Therapy, Faculty of Associated Medical Sciences, Chiang Mai University, Chiang Mai 50200, Thailand; siripat_khammesri@cmu.ac.th; 2 Department of Radiologic Technology, Faculty of Associated Medical Sciences, Chiang Mai University, Chiang Mai 50200, Thailand; 3 Elephant Hospital, National Elephant Institute, Forest Industry Organization, Lampang 52190, Thailand; kittikul.nam@gmail.com (K.N.); magiemeema@gmail.com (N.K.); pichamoneye@gmail.com (P.U.); 4 Center of Elephant and Wildlife Health, Animal Hospital, Faculty of Veterinary Medicine, Chiang Mai University, Chiang Mai 50100, Thailand; chatchote.thitaram@cmu.ac.th (C.T.); brownjan@si.edu (J.L.B.); 5 Faculty of Veterinary Medicine, Chiang Mai University, Chiang Mai 50100, Thailand; 6 Elephant, Wildlife and Companion Animals Research Group, Chiang Mai University, Chiang Mai 50100, Thailand; 7 Center for Species Survival, Smithsonian National Zoo and Conservation Biology Institute, 1500 Remount Rd., Front Royal, VA 22630, USA

**Keywords:** elephant, gait analysis, vertical asymmetry, signal processing

## Abstract

The early detection of lameness in elephants is essential for effective treatment and welfare enhancement, but subtle gait abnormalities are often difficult to identify visually. This study aimed to evaluate vertical movement symmetry in Asian elephants using cross-correlation analysis of data from inertial measurement units (IMUs). Six elephants were assessed, including individuals with normal gait and one exhibiting an abnormal gait. IMU sensors were attached to the proximal and distal segments of the forelimbs and hindlimbs to record vertical movements during straight-line walking. Cross-correlation coefficients were calculated to quantify the symmetry between the left and right limbs, providing an objective measure of gait patterns. This method provided an objective assessment of gait patterns and demonstrated potential in detecting lameness in elephants. This approach could facilitate the early identification of gait abnormalities, enabling timely interventions and potentially improving treatment outcomes and the welfare of captive elephant populations. Further studies involving a larger number of elephants with confirmed gait abnormalities are necessary to validate the robustness and reliability of this approach.

## Simple Summary

Lameness in elephants can significantly impact their health and welfare, but early detection remains challenging due to the subtle initial signs. This study investigated the use of inertial measurement units (IMUs) to objectively assess gait symmetry in Asian elephants. By attaching IMUs to the limbs of six Asian elephants, we measured vertical movement patterns during walking. The aim was to determine vertical movement with these sensors in identifying gait abnormalities. This non-invasive and objective approach may assist veterinarians in identifying gait problems early, leading to timely treatments and improved welfare for elephants in captivity. To fully validate this technology, future studies should include a larger number of elephants with confirmed gait abnormalities diagnosed by experienced veterinarians to ensure its robustness and reliability.

## 1. Introduction

Accurately identifying lameness in animals is essential for reliable diagnosis and assessment of treatment efficacy. However, in cases of mild lameness, localizing the condition to the affected limb can be challenging due to subtle compensations that are difficult to detect visually [1,2]. Traditionally, gait assessment in clinical settings relies on subjective evaluations, where veterinarians observe the animal’s movement to assess gait conformity and symmetry. These visual inspections depend on the observer’s experience, which can lead to disagreements among practitioners regarding the level of lameness assigned to a single animal. While subjective assessments are common, objective techniques for identifying and measuring lameness minimize the risk of evaluation bias [3]. Objective approaches quantify lameness on a continuous scale, offering greater precision than subjective lameness scales that categorize the condition into discrete categories. By providing more nuanced and accurate measurements, objective methods enhance the ability to detect subtle changes in gait, potentially leading to earlier intervention and more effective treatment strategies.

For larger animals like elephants, previous studies have attempted to apply technological solutions such as pressure-sensitive walkways or video motion capture to assess gait [4,5,6]. Force plates and pressure-sensitive walkways are challenging to construct for elephants because of their size, weight, and environmental requirements [5]. Space and lighting limitations limit the use of video motion capture in field settings, despite its effectiveness in controlled settings [7]. On the other hand, IMUs are small and light and can be used to gather detailed kinematic data in the field, as shown by research with horses [8]. The features mentioned previously make IMUs particularly suitable for handling the complicated challenges of elephant gait analysis. Three-dimensional kinematic analyses of Asian elephants have emphasized joint angle variations and limb coordination [7,9,10], contributing to the understanding of the mechanical strategies elephants use to support their massive bodies during movement. Microelectromechanical systems (MEMSs) and inertial measurement units (IMUs) have proven to be effective for the biomechanical evaluation of gait kinematics and various locomotion types in animals, including dogs [11,12,13], horses [1,8,14], and elephants [15,16]. Objective gait analysis in horses has been successfully performed using body-mounted inertial sensors. Keegan et al. [8,17] validated the efficacy of IMUs in identifying lameness in horses, demonstrating that these devices provide reliable and quantitative information on gait asymmetries, thereby improving diagnostic accuracy. Given the challenges associated with traditional gait analysis methods in elephants, IMUs offer a promising alternative due to their portability, affordability, and ability to capture detailed kinematic data in various environments [16]. Moreover, IMUs produce data on gait variables comparable to those obtained through force plate methods, pressure walkways, or video recording techniques [14,18,19].

Kinematic parameters derived from IMUs have demonstrated utility in detecting compensatory mechanisms in animals with lameness. For example, vertical movement asymmetries such as head movement asymmetry in response to forelimb lameness and pelvic asymmetry for hindlimb lameness have been used as indicators of gait abnormalities in both small and large animals, including in dogs [13,20] and horses [21,22,23]. Variations in the vertical movement of body segments are associated with lameness, providing a non-invasive method to detect gait abnormalities. In elephants, assessing vertical movement patterns may offer valuable insights into movement asymmetry that could help practitioners locate and identify gait problems, which could promote better health and welfare. However, despite the availability of various tools in gait analysis, there is limited research on the use of IMUs to assess vertical movement symmetry in elephants.

Therefore, we hypothesize that elephants with normal gait will exhibit high symmetry in vertical limb movements. This study aims to evaluate the vertical movement symmetry between normal and abnormal gait patterns in Asian elephants using cross-correlation coefficients. By comparing movement signals from the left and right sides, this study explores the utility of cross-correlation coefficients as an IMU-derived variable to represent gait abnormality.

## 2. Materials and Methods

### 2.1. Gait Assessment

Visual gait assessments were conducted by three veterinarians with over five years of professional experience in elephant healthcare. The assessments were based on the agreement of all three assessors. The Gait Score system, ranging from 0 to 2 [24], with 0 indicating the absence of visible lameness or limb abnormalities, 1 indicating mild lameness or subtle alterations in gait, and 2 indicating severe lameness characterized by evident gait abnormalities or limb deformities, including an unwillingness to bend, flex, or bear weight on the affected limb.

### 2.2. Animals

This study evaluated six Asian elephants (*Elephas maximus*) (1 male, 5 females), consisting of five with normal movement (N1–N5, Gait Score 0 out of 2) and one with an abnormal gait characterized by circumduction (A1, Gait Score 2 out of 2). The elephants ranged in age from 15 to 54 years, with a body condition score of 3.6 ± 0.6 (range 3–4.5) out of 5 and an average shoulder height of 239 ± 13 cm (range 220–256 cm). The demographic data for each elephant are summarized in Table 1. All elephants were housed at the National Elephant Institute, Forest Industry Organization, Thailand.

Elephants N1, N4, and N5 participated in daily shows lasting no more than 40 min, occurring twice per day. Elephants N2 and N3 worked as riding elephants with a saddle, covering distances of approximately 800 m per session, lasting no more than 30 min, and occurring 2–3 times per day. Elephant A1, the lame elephant, was a 31-year-old male with a body condition score of 3.5 and did not participate in any tourist activities.

During non-working hours, the elephants were tethered on a 3 m chain under a shaded structure, allowing trunk-length physical access to other elephants. At night, they were tethered in a designated resting area with a minimum 5 m chain from 1700 h to 0600 h. The elephants were fed primarily Bana grass (*Pennisetum purpureum X*, *P*. *americanum hybrid*) and supplements such as bananas and sugar cane, with free access to fresh water. All animal procedures were approved by the Institutional Animal Care and Use Committee, Faculty of Veterinary Medicine, Chiang Mai University, Chiang Mai, Thailand (license number S18/2567).

### 2.3. IMU System

**Figure 1:**
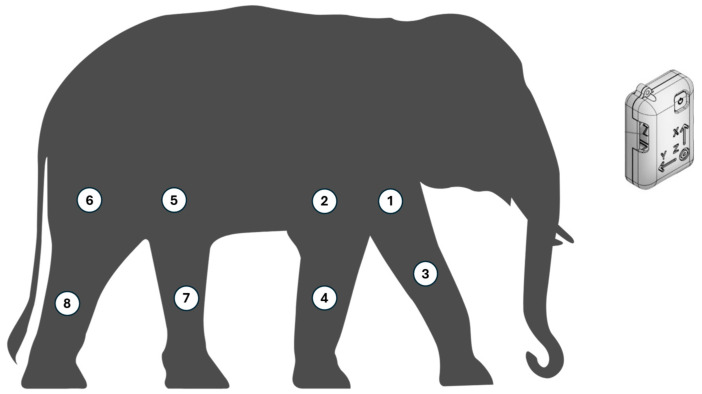
The position of the eight sensors mounted to an elephant’s body.

Data were collected using eight inertial measurement unit (IMU) sensors (STT Ingeniera Y Sistemas, San Sebastián, Spain), each comprising an accelerometer, gyroscope, and magnetometer, sampling at 50 Hz. The sensors were securely attached to the skin overlying the midshaft of the humerus (proximal forelimb), radius (distal forelimb), femur (proximal hindlimb), and tibia (distal hindlimb) using hypoallergenic adhesive tape to minimize movement artifacts. Sensor alignment was checked visually and adjusted as necessary. Figure 1 illustrates the sensor placement on an elephant.

The IMU system recorded six degrees of freedom, capturing acceleration outputs relative to local axes within global coordinate systems. Sensor signals were transmitted wirelessly via Wi-Fi to a software interface, which helped minimize the effects of initial acceleration and ending deceleration on the data. Data collection was conducted through four consecutive trials for each elephant, and data from the three trials with the most consistent walking patterns were selected for analysis. Consistency was assessed based on visual observation of gait with a steady pace during the trials to ensure reliable data.

### 2.4. Data Collection Protocol

In Asian elephants, moving slowly reduces the vertical movement of the center of mass (COM). At slow speeds, around 1.5 m/s, this vertical movement remains under 0.03 m and decreases with increasing speed [6]. The restricted vertical movement at slow speeds promotes stability, supporting the detection of subtle gait abnormalities, crucial for identifying asymmetries and focus on identifying minor gait abnormalities by assessing vertical movement symmetry.

**Figure 2:**
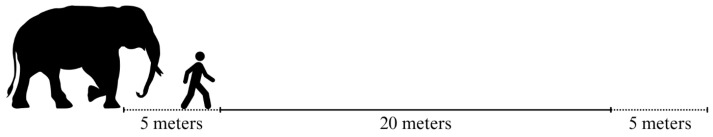
Schematic of the data collection protocol. The total distance was 30 m, divided into 20 m of data collection and 5 m at the beginning and end to minimize the effects of acceleration and deceleration.

The trials were performed with the elephants walking on hard ground (concrete and asphalt) over a level, straight stretch of 20 m (Figure 2). Several practice trials were conducted to ensure familiarity with the experimental setup and encourage a natural walking pace. Elephants were guided by their mahout using positive reinforcement techniques, such as food rewards and verbal commands, to maintain a steady pace of 1–2 m/s. At slow speeds, the hip and shoulder joints reach a peak (rise and then fall) during mid-stance [10]. To ensure a consistent walking speed, the elephants walked a minimum of 5 m before and after the data collection zone to allow for acceleration and deceleration phases, which a previous study on limb movement found to be significant (16).

### 2.5. Signal Processing

**Figure 3:**
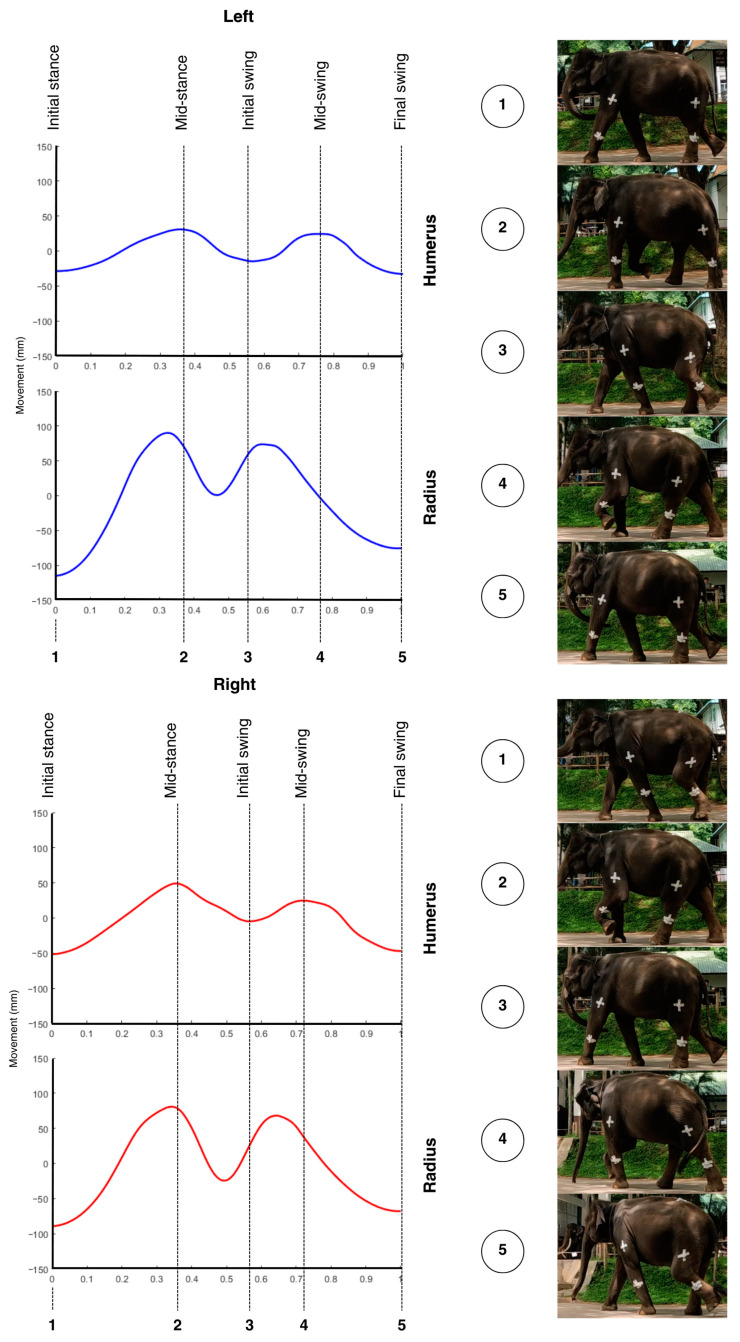
Average vertical movement signal for the left (blue) and right (red) forelimbs, with phases of the gait cycle (initial stance, mid-stance, initial swing, mid-swing, final swing) marked by vertical lines. The lowest point at the beginning of each cycle represents the initial stance phase, where the gait cycle commences. Photographic snapshots of each phase are displayed below, illustrating the corresponding limb positions throughout the cycle.

**Figure 4:**
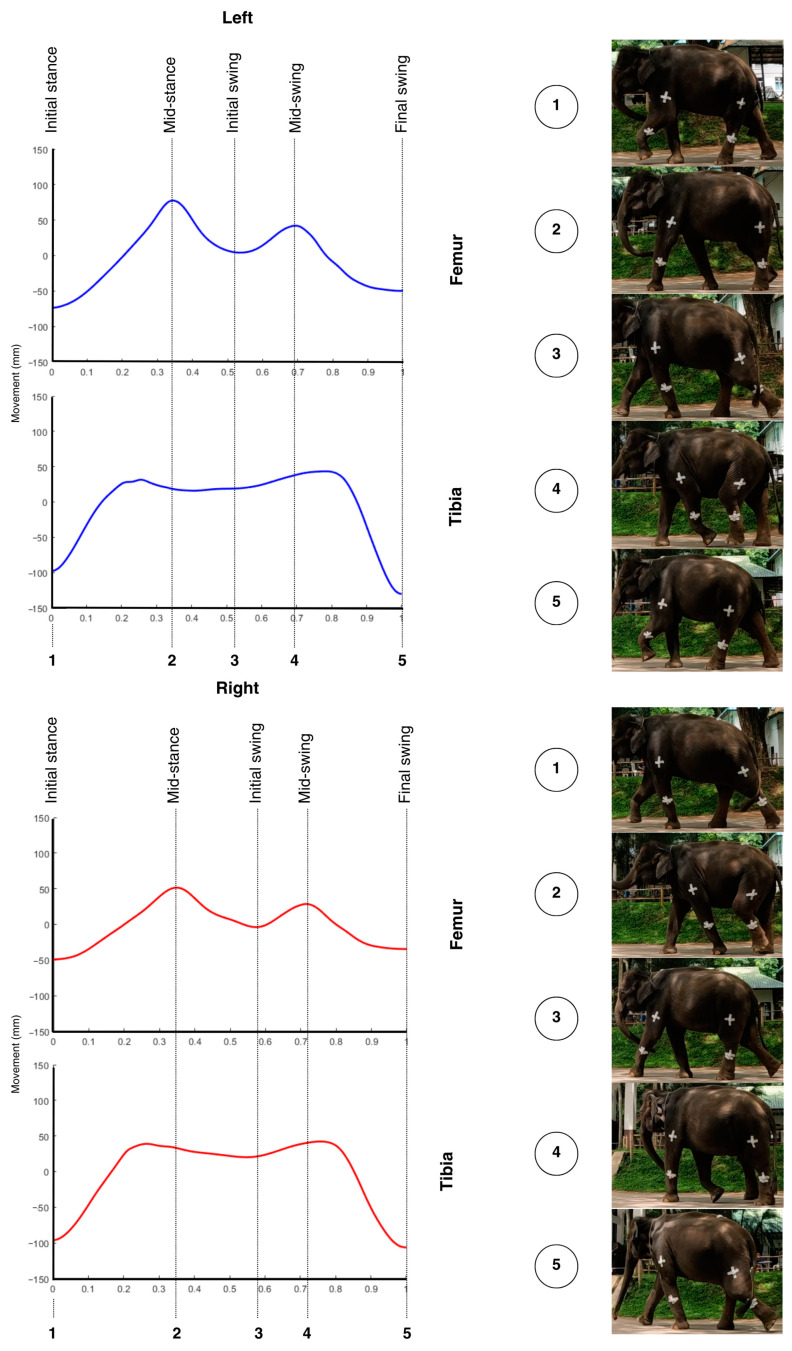
Average vertical movement signal for the left (blue) and right (red) hindlimbs, with phases of the gait cycle (initial stance, mid-stance, initial swing, mid-swing, final swing) marked by vertical lines. The lowest point at the beginning of each cycle represents the initial stance phase, where the gait cycle commences. Photographic snapshots of each phase are displayed below, illustrating the corresponding limb positions throughout the cycle.

Signal processing was conducted by measuring vertical movements during five consecutive gait cycles selected from the middle of each walking session. The data were imported into custom MATLAB software, which processed the vertical acceleration signals from the IMUs attached to the proximal and distal segments of the forelimbs and hindlimbs. The filtered acceleration data with a low-pass Butterworth filter [25,26] were double-integrated to obtain displacement over time, converting the results to millimeters. To account for potential velocity-related variations, the vertical displacement data were normalized to a range of 0–1 before applying cross-correlation analysis. This normalization ensured that differences in walking speed did not influence the comparative analysis of movement symmetry. The starting point of each gait cycle was identified at the lowest point during the cycle, corresponding to the initial stance phase; examples for the forelimb and hindlimb are shown in Figure 3 and Figure 4, respectively.

**Figure 5:**
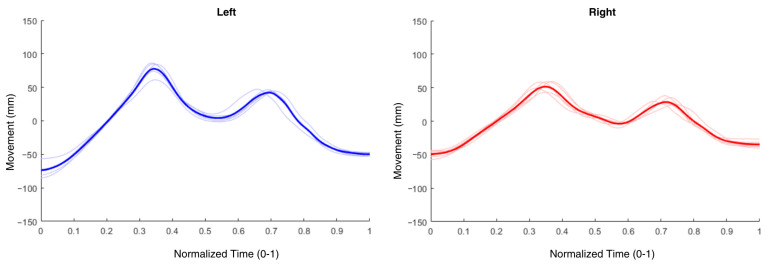
Average vertical movement signal for the left (blue) and right (red) sides, obtained by averaging five normalized gait cycles for each limb. This visualization highlights the typical vertical movement patterns across cycles, enabling the assessment of symmetry between the left and right limbs.

Each cycle was normalized to a common time frame to ensure consistent comparison across cycles. Average vertical movement signals for the left and right sides were generated by averaging the five normalized cycles for each side (Figure 5).

In signal processing, cross-correlation analysis [27,28] is widely recognized as an effective method for detecting correlations between two signals. This technique allows for the assessment of similarity between two time-series signals as they evolve over time, offering the sensitivity needed to identify subtle phase shifts. These characteristics make cross-correlation particularly useful for evaluating signal behaviors and assessing synchronization.

Cross-correlation calculates the similarity between the vertical displacement signals from the left and right limbs as a function of time lag. According to Buck et al. [29], the cross-correlation function $R_{xy}(\tau )$ for two signals, where x(t) represents the left limb’s vertical movement and y(t) represents the right limb’s vertical movement, is defined by:

$$R_{xy}(\tau )=\int_{-\infty }^{\infty }x(t)y(t+\tau )\;dt$$

where *τ* represents the time lag applied to one of the signals. By shifting one signal relative to the other and calculating the correlation at each lag, this function provides insight into the degree of alignment or symmetry between the left and right movements. High values of $R_{xy}(\tau )$ at specific lags suggest strong correlation or synchronization between the limbs, while lower values indicate asymmetry, which can be interpreted as compensatory movement or deviations from normal gait patterns.

In practice, this analysis is implemented discreetly, as described by Stoica and Moses [30], using the formula:

$$R_{xy}[k]=\sum_{n=0}^{N-1}x[n]\cdot y[n+k]$$

where k is the discrete time lag and N represents the number of data points in each trial. Normalizing these cross-correlation coefficients yields values within the range of $\left[-1,1\right]$, where a value close to 1 indicates high symmetry in vertical movement, suggesting normal gait, and values significantly lower than 1 suggest asymmetries, which may indicate gait abnormalities.

In this study, cross-correlation analysis was applied to compare the vertical movement signals of the left and right limbs of elephants during gait analysis. This technique provides a robust method to evaluate the degree of symmetry between the two signals, allowing for the detection of subtle phase shifts and similarities in movement patterns, which can indicate gait abnormalities. To measure the symmetry between sides, cross-correlation analysis [26] was then performed on these average profiles to measure gait symmetry for each sensor pair in proximal and distal segments.

## 3. Results

This study evaluated gait symmetry in Asian elephants by analyzing vertical movement cross-correlation coefficients across the proximal and distal segments of the forelimb and hindlimb bones. The sample comprised six elephants: five with normal gait (N1, N2, N3, N4, and N5, each assigned a Gait Score of 0 out of 2) (Table 2) and one with an abnormal gait (A1, with a Gait Score of 2 out of 2) (Table 3). Cross-correlation coefficient values represent symmetry between the left and right sides over five consecutive gait cycles within a trial, and standard deviations (SDs) were calculated from three selected trials for each subject.

For the five elephants with normal gaits, the mean vertical movement cross-correlation coefficients and standard deviations varied across limbs and segments. In the forelimb proximal segments (humerus), the mean cross-correlation coefficients ranged from 0.77 ± 0.00 (N3) to 0.93 ± 0.03 (N1), while the distal segments (radius) showed values from 0.75 ± 0.09 (N4) to 0.98 ± 0.01 (N5). In the hindlimb proximal segments (femur), high symmetry was observed, with mean cross-correlation coefficients ranging from 0.70 ± 0.01 (N4) to 0.99 ± 0.01 (N1), while the distal segments (tibia) ranged from 0.83 ± 0.12 (N4) to 0.98 ± 0.03 (N5).

The elephant with an abnormal gait (A1) exhibited notably lower cross-correlation coefficients in most segments, indicating reduced symmetry in movement. The forelimb proximal segment showed a mean cross-correlation coefficient of 0.48 ± 0.05, and the distal segment was 0.57 ± 0.12. In the hindlimb, the proximal segment had a cross-correlation coefficient of 0.55 ± 0.04, while the distal segment was relatively higher at 0.92 ± 0.01.

**Figure 6:**
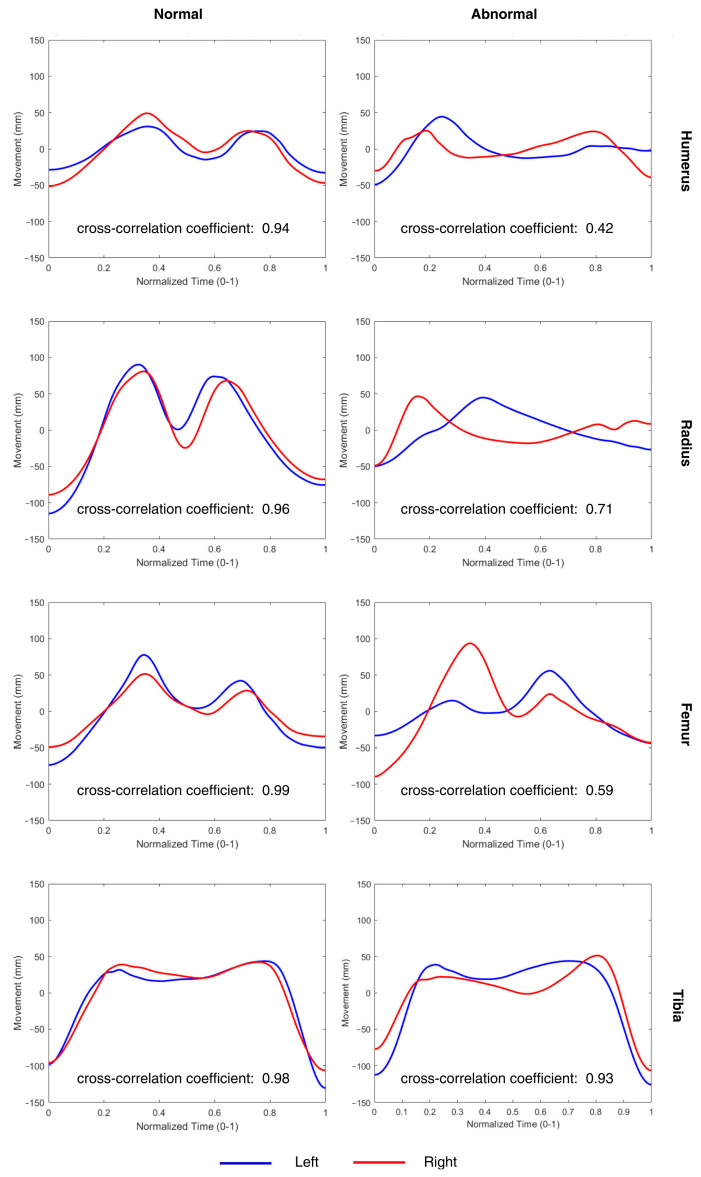
Average vertical movement comparison for an elephant with a normal gait (**left** column) and an elephant with an abnormal gait (**right** column) in the proximal and distal segments of the forelimb and hindlimb. Cross-correlation coefficients are shown for each segment, reflecting the symmetry in vertical displacement between the left (blue) and right (red) sides.

Figure 6 represents the average vertical movement comparison between the left (blue) and right (red) sides in a normal-gait elephant (N1) and the abnormal-gait elephant (A1), illustrating vertical movement patterns in both the proximal and distal segments of the forelimb and hindlimb. In the normal-gait elephant, the observed symmetry across limb segments is reflected in high cross-correlation coefficients. In contrast, the abnormal-gait elephant, which has a left forelimb issue with stiffness along the limb and exhibits a circumduction gait, shows lower cross-correlation coefficients, indicating reduced symmetry. These findings highlight the lack of symmetry in vertical movement across limb segments in the abnormal-gait elephant, suggesting compensatory adjustments in response to gait abnormalities.

The elephant with an abnormal gait (A1) exhibited significantly lower cross-correlation coefficients in the proximal segments of both the forelimb and hindlimb compared to the normal-gait elephants. This reduction in symmetry is particularly notable in the forelimb, which corresponds with the observed clinical signs of stiffness and circumduction in the left forelimb. The relatively higher coefficient in the distal hindlimb segment suggests that the hindlimb was less affected or that compensatory mechanisms were more effective in that segment.

## 4. Discussion

This study evaluated the vertical movement patterns in the proximal and distal segments of the forelimbs and hindlimbs of Asian elephants. By employing cross-correlation coefficient analysis, we assessed the temporal similarity and phase relationships between time-series signals from the left and right sides, effectively measuring symmetry in bilateral limb movements during walking. Cross-correlation quantifies the correlation between two signals at various time intervals, allowing for the investigation of similarities and phase shifts [26,29,30]. When signal variations align across time lags, cross-correlation helps to quantify asymmetries or compensatory patterns, which are crucial for detecting gait abnormalities. The findings demonstrated that changes in vertical movement symmetry, assessed through cross-correlation coefficients, effectively differentiated between normal and abnormal gait patterns in these elephants.

In this study, elephants with normal gaits exhibited high cross-correlation coefficients in vertical movement between the left and right limbs, indicating strong symmetry. In contrast, the elephant with an abnormal gait showed notably lower cross-correlation coefficients, especially in the forelimb segments. The lower cross-correlation coefficients observed in the abnormal elephant suggest significant asymmetry in limb movements, likely due to compensatory mechanisms arising from discomfort or injury. Injury or discomfort in a limb can affect an elephant’s weight-bearing, leading to uneven load distribution and adjustments in stride length or joint angles. These compensatory movements result in measurable asymmetries in vertical limb movement. These results support our hypothesis and demonstrate that vertical movement symmetry is a sensitive parameter for detecting lameness in elephants. Horses and elephants, despite differences in size and locomotion, both exhibit vertical movement asymmetries when experiencing lameness. In horses, such asymmetries have been linked to altered head and pelvic movements [8,17]. Similarly, our findings suggest that elephants compensate for discomfort in a limb by adjusting their gait patterns, resulting in detectable asymmetries. This could help practitioners to identify lameness early, enabling timely treatment options and potentially enhancing the welfare of captive elephant populations.

In summary, this preliminary study analyzed gait patterns in six Asian elephants, including five with normal gaits and one with an abnormal gait. While the results provide valuable insights into vertical movement symmetry and its relationship to gait abnormalities, the small sample size and the inclusion of only one elephant with an abnormal gait limit the generalizability of the findings. As such, these results should be interpreted with caution, and further research is needed to validate and extend these findings to a broader population. Expanding the sample size to include more elephants with various gait patterns would enhance the generalizability and provide a more comprehensive understanding of how vertical movement asymmetry correlates with various types of lameness or compensatory movements. Moreover, longitudinal studies that monitor changes in gait patterns over time through cross-correlation analysis, particularly before and after rehabilitation or treatment, could further support clinical applications. Such studies would allow practitioners to assess intervention effectiveness, detect gradual improvements, and monitor compensatory adjustments, offering early indicators of treatment success. This technology is accessible and can be utilized in field conditions common in elephant management. While our study focused on vertical movement symmetry, other factors, such as gait kinematics, could also influence gait patterns. Future studies incorporating these variables may provide a more comprehensive understanding of elephant locomotion.

## 5. Conclusions

This pilot study suggests that cross-correlation coefficients derived from IMU signals may offer a useful approach for differentiating between normal and abnormal gait patterns in Asian elephants. The findings highlight the potential of this method as a diagnostic tool for identifying gait abnormalities. However, further research with larger sample sizes and a variety of lameness conditions is necessary to validate its reliability and applicability in clinical settings.

## Figures and Tables

**Table 1:** Demographic data of the elephants used in this study.

Elephant	Sex	Age (Years)	Gait Score	Body Mass (kg)	Shoulder Height (cm)	Body Condition Score	Type of Work
N1	F	20	0	2620	245	3	Show
N2	F	48	0	3015	232	4.5	Riding on saddle
N3	F	54	0	3615	245	3	Riding on saddle
N4	F	15	0	2035	220	3.5	Show
N5	F	18	0	2590	234	4	Show
A1	M	31	2	4120	256	3.5	No working

**Table 2:** Mean (±SD) cross-correlation coefficients of vertical movement between left and right forelimbs and hindlimbs in five normal-gait elephants (N1–N5).

			Trial_1	Trial_2	Trial_3	Mean	SD
N1	Forelimb	Proximal (Humerus)	0.90	0.94	0.95	0.93	0.03
		Distal (Radius)	0.98	0.96	0.97	0.97	0.01
	Hindlimb	Proximal (Femur)	0.99	0.99	0.98	0.99	0.01
		Distal (Tibia)	0.98	0.98	0.93	0.96	0.03
N2	Forelimb	Proximal (Humerus)	0.84	0.96	0.77	0.86	0.10
		Distal (Radius)	0.72	0.79	0.74	0.75	0.03
	Hindlimb	Proximal (Femur)	0.99	0.98	0.98	0.98	0.00
		Distal (Tibia)	0.96	0.96	0.99	0.97	0.02
N3	Forelimb	Proximal (Humerus)	0.77	0.77	0.76	0.77	0.00
		Distal (Radius)	0.86	0.72	0.85	0.81	0.08
	Hindlimb	Proximal (Femur)	0.91	0.95	0.89	0.92	0.03
		Distal (Tibia)	0.93	0.96	0.88	0.92	0.04
N4	Forelimb	Proximal (Humerus)	0.86	0.96	0.92	0.91	0.05
		Distal (Radius)	0.67	0.73	0.85	0.75	0.09
	Hindlimb	Proximal (Femur)	0.71	0.70	0.70	0.70	0.01
		Distal (Tibia)	0.94	0.71	0.82	0.83	0.12
N5	Forelimb	Proximal (Humerus)	0.71	0.81	0.86	0.79	0.07
		Distal (Radius)	0.98	0.99	0.98	0.98	0.01
	Hindlimb	Proximal (Femur)	0.71	0.77	0.88	0.78	0.09
		Distal (Tibia)	0.94	1.00	1.00	0.98	0.03

**Table 3:** Mean (±SD) cross-correlation coefficients of vertical movement between left and right forelimbs and hindlimbs in an abnormal gait (A1) based on a visual Gait Score.

		Trial_1	Trial_2	Trial_3	Mean	SD
Forelimb	Proximal (Humerus)	0.49	0.42	0.51	0.48	0.05
	Distal (Radius)	0.48	0.71	0.52	0.57	0.12
Hindlimb	Proximal (Femur)	0.55	0.59	0.52	0.55	0.04
	Distal (Tibia)	0.91	0.93	0.91	0.92	0.01

## Data Availability

The data analyzed for this study are available from the corresponding author upon reasonable request.
